# Spatial evaluation of the accessibility of public service facilities in Shanghai: A community differentiation perspective

**DOI:** 10.1371/journal.pone.0268862

**Published:** 2022-05-26

**Authors:** Juan Wang, Jun Zhou

**Affiliations:** 1 College of Urban Construction, Zhejiang Shuren University, Hangzhou, China; 2 International Science and Technology Cooperation Base of Zhejiang Province: Remote Sensing Image Processing and Application, Hangzhou, China; 3 School of Design and Architecture, Zhejiang University of Technology, Hangzhou, China; Tongji University, CHINA

## Abstract

Following the ‘people-oriented’ concept, increased attention should be paid to the heterogeneity of community residents when configuring public service facilities. Taking Shanghai as an example, this study analyzed the spatial pattern of urban and rural community differentiation and evaluated spatial differences in the level of accessibility to four types of public service facilities based on the shortest travel distance. There were significant differences between community types in Shanghai, with a clear circular structure in the urban and rural spaces. Here, facility accessibility decreased from the core to the periphery. Population density, income level, and the shortest walking distance to facilities revealed a significant negative correlation. Age level, household registration, and the shortest walking distance to facilities were partially weakly correlated or uncorrelated. The spatial matching of population density and facility accessibility simultaneously presented a circular pattern and heterogeneity, while planned new towns in suburbs and mid-suburbs presented a double-medium-sized match of the same community type. From the perspective of community differentiation, mechanisms behind the formation of the spatial pattern of facility accessibility included urban-rural pattern continuity and differences in allocation specifications, the continuity of population agglomeration and the accumulation of facility construction, and urban planning guidance and population agglomeration.

## 1. Introduction

China is in a rapid stage of urbanization. With the rapid expansion of the urban scale, the allocation of urban service facilities is gradually improving, but it will inevitably bring problems associated with the urban social space as well. At the same time, the advancement of new urbanization puts higher demands on cities. China’s urbanization has changed from an extensive development stage focusing on quantity and scale to a development stage focusing on the improvement of quality connotation. The CPC Central Committee and the State Council proposed in 2017 to ‘strive to improve the community service capacity and level, gradually realize the full coverage of urban and rural community comprehensive service facilities, improve the utilization rate of urban comprehensive service facilities, and promote the accuracy of community services’ to strengthen and improve urban and rural community governance in 2017. How to provide community public services more efficiently has become a universal problem in community governance, affecting the life perception of urban residents and the formulation of urban development strategy. At present, the main social contradiction in China has been transformed into the contradiction between the people’s growing needs for a better life and imbalanced and insufficient development. There is an obvious gap between urban and rural regional development. There is an imbalance and insufficient allocation of public services such as education, medical treatment, and pension, which cannot meet the demand of residents for community public services. However, for a long time, the allocation standard of community public service facilities that have followed the ‘graded matching’ and ‘thousand person index’ has failed to fully consider the community heterogeneity accompanied by the rapid growth of population scale in mega cities, ignored the differentiated needs of different types of community residents, and there is a general mismatch between the supply of community public service facilities and the needs of residents. It has a negative impact on the convenience of daily life of residents and the sense of belonging to a community, resulting in the gradual erosion of the original intention of ‘community’. In large cities, issues of the differentiation between communities are generally objective and persistent. In this regard, changes in the allocation standards for community public service facilities highlight the need for accessibility and concerns regarding heterogeneity of residents [1,2]. In this context, it is also necessary to study the attribute differences of community residents in the research on the allocation of public service facilities, so as to provide differentiated and accurate allocation.

During periods of rapid growth in the mega city context, urban and rural residents choose different locations and public service levels of community agglomeration. This creates heterogeneity between urban and rural communities in regard to several socioeconomic attributes, including age structure, registered residence structure, and income level [3–5]. There is a long history of research on community differentiation from scholars in both the East and West. Focusing on location theory and the socioeconomic attributes of residents, Western scholars have used location, race, income level, and other factors to divide urban and suburban communities [6], while Chinese scholars have divided communities into types based on material elements and resident characteristics, including community location, construction age, registered residence ratio, housing price, age structure, and population density [7–10].

At the same time, there is a direct relationship between the way public service facilities are allocated and the residential quality of life. For this reason, facility accessibility has remained a central research topic in urban and rural planning, geography, and other disciplines. Several Western scholars have explored the connections between facility accessibility and the socioeconomic attributes of residents [11–15]. Many case studies have shown that spatial differences in the level of accessibility to public service facilities affect both social equity and justice utility. Chinese scholars have recently begun to emphasize the need to ensure that public facility accessibility reflects the socioeconomic attributes of residents. For example, Jiang, Zhou, and Xiao analyzed a phenomenon in which Guangzhou park green spaces migrated to the high income class [16]. Tang and Gu discussed a potential spatial mismatch between the distribution of public green space resources and the permanent population in the central urban area of Shanghai [17]. Tian et al. compared and analyzed a spatial deprivation phenomenon in local and foreign community facilities in the fringe area of Shanghai central city [18]. Zeng, Xiang, and Zhang analyzed the accessibility of community service facilities in Nanjing to better understand spatial deprivations among urban low-income groups [19]. Xu, Zhang, and Chen explored whether facility accessibility matched the needs of vulnerable groups in Beijing [20]. Ding et al. developed spatial and social equity models to evaluate the social equity of the distribution of public elementary schools in downtown Hangzhou [21]. Meanwhile, some scholars have analyzed the influencing factors and mechanisms behind the matching patterns that arise between facility accessibility and the socioeconomic characteristics of residents [22]. Generally speaking, this type of research is primarily focused on issues that affect vulnerable groups (e.g., migrant populations and low-income groups), with basic analysis units including subdistricts, residential lands, neighbourhood committees, and regions. However, the scope of case cities is mostly partial or complete urban areas, and there is a lack of research on a larger scale for urban and rural spaces.

The above research on community differences shows that the differentiation of community types in big cities is objective and will exist for a long time, but the community type classification facing the subdistricts level is rare. The subdistrict level integrates sociological concepts and China’s unique administrative attributes and is consistent with the spatial unit of community planning in Shanghai. Therefore, this paper selects the subdistrict level as the community research category, and the research scope also breaks through the urban areas where the above research is concentrated, but faces the overall urban and rural space on a larger scale.

The selection of the subdistrict level for community type division is consistent with the current administrative unit division in regional scope, which has guiding significance for the spatial layout and implementation of public service facilities. Choosing the scope of Shanghai is helpful in objectively understanding the differences in the allocation of public service facilities between urban and rural areas. Therefore, this paper uses the relevant theories and methods of urban and rural planning, urban sociology, urban geography, and geographic information science, selects community differentiation as the research entry point, makes a spatial evaluation on the accessibility of community public service facilities, and deeply explores the spatial matching between the accessibility of community public service facilities and community social attributes in urban and rural areas of Shanghai.

Using Shanghai as a representative case, this study took the subdistrict unit as the division of community type to analyze the spatial pattern of community differentiation based on the superposition of socioeconomic attributes and spatial attributes, then analyzed the spatial differentiation characteristics of accessibility to four types of public service facilities based on the shortest network path distance. From the perspective of community differentiation, this study analyzed the mechanism behind the formation of spatial patterns in facility accessibility.

## 2. Study area and data sources

### 2.1. Research scope and regional division

**Fig 1:**
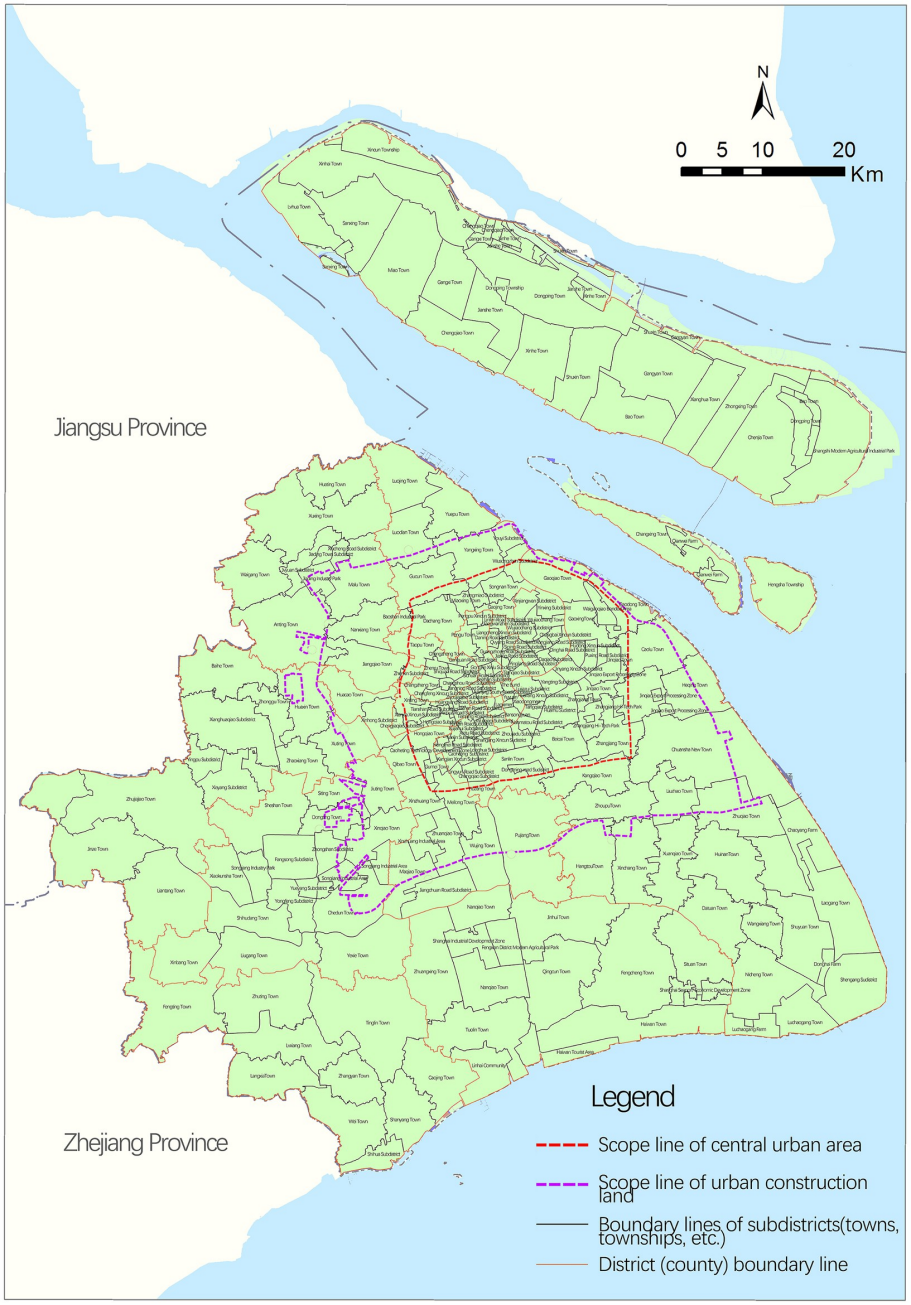
Division of study region. Source: Created by the author based on the base map of Shanghai from the Open Street Map(OSM) geographic data platform(https://www.openstreetmap.org/).

The research scope was Shanghai, with the subdistrict (e.g., towns and townships) used as the basic unit of analysis (a total of 230 taken from the ‘Sixth census’ data). Based on the theory of urban spatial structure and Shanghai planning practices, Shanghai is divided into three regions (Fig 1), including the central city, marginal area, and peripheral area [6]. The outer ring road in Shanghai was taken as the scope line of the central city, while the scope line of urban construction land defined in the previous round of the Shanghai urban master plan was taken as the boundary between the marginal area and periphery.

### 2.2. Data source and processing

The research data mainly consisted of three parts, including basic map data, community attribute statistics, and public service facilities statistics. The basic map is the administrative division map of Shanghai; the base map was taken from spatial data obtained via the ‘Sixth census’. The urban pedestrian road network was imported and processed based on open street map data, including urban trunk road, secondary trunk road, branch road, and residential group road. Data on population density, age composition, and registered community residence status were taken from the ‘Sixth census’. Income level was analyzed based on housing price data from the Anjuke website (https://shanghai.anjuke.com/).

Against the background of high aging rates in Shanghai, we selected facilities that were closely related to daily residential life and had flexible service scopes, including cultural facilities, medical facilities, farmer’s markets, and parks. For facility projects, we selected community level and public service facilities with high frequencies, which can provide high-quality and high-level public service facilities for community residents. Information on various facilities was subject to the data published on the official website and verified by sampling surveys and telephone calls. Among them, cultural facilities included community level and above libraries and cultural centres, with a total of 271 library institutions and 265 cultural centres. The statistical caliber of medical facilities was 371 public general hospitals of level I, II, and III. The total number of farmer’s markes was 976, including those that were in operation and that were to be slated for renovation. The statistical calibre of public green space was the officially published park. The park directory showed a total of 234 parks. We simplifies the analysis at the municipal level and extracted the centroid of various service facilities based on their spatial locations. Table 1 shows a detailed list of data types and sources.

**Table 1:** Data list with sources.

Data type	Data list	Data source
Basic map data	Urban administrative zoning map	‘Sixth census’ data; OpenStreetMap
	Spatial location within traffic network	
Statistical data for community attributes	Population density	‘Sixth census’ data
	Age	
	Registered residence structure	
	Income level	Housing price data from the Anjuke website
Statistical data for public service facilities	Cultural facilities	Shanghai Municipal Administration of Culture, Radio, Film and Television; official website of the Shanghai Central Library; Gaode POI data
	Medical facilities	Shanghai Health and Family Planning Commission; Gaode POI data
	Farmer’s markets	Shanghai Municipal Commission of Commerce website
	Parks	Shanghai Greening and City Appearance Administration website; Gaode POI data

## 3. Spatial pattern of community differentiation in Shanghai

### 3.1. Division of community socioeconomic attributes

We selected four socioeconomic attributes (i.e., population density, registered residence structure, age structure, and income level) that were directly related to community governance [6]. According to population density, the community was divided into two types, including high density and general density. The household registration structure was based on the proportion of registered residents in the permanent population. As such, the community was divided into three types, including local population (the community with the household registration as the main population), mixed population, and foreign population (the community with a predominantly non-registered permanent population). For age structure, we took the proportion of the elderly population within the total population as the index, then divided the community into aging and non-aging groups. Based on the average price of community housing, income level was used to divide the community into three types, including high-income, middle-income, and low-income (Table 2).

**Table 2:** Division and grading standard of community socioeconomic attributes in Shanghai.

Socioeconomic attributes	Indicators	Division (Grading standard)		
Age structure	People over 60 years old/total population	Non-aging (<10%)	Aging (≥10%)	
Population density	People/ km^**2**^	General density (<15000)	High density (≥15000)	
Income level	Housing prices (yuan/ m^2^)	Low-income (<15000)	Middle-income (15000–35000)	High-income (≥35000)
Registered residence structure	Registered residents / total population	Foreign population (<30%)	Mixed population (30%-70%)	Local population (≥70%)

### 3.2. Designations of community type samples

To describe and analyze the spatial pattern of community type differentiation in Shanghai, the community types were given alphanumeric designations. The encoding rule was: ‘age structure—population density—income level- registered residence structure’, with each designation corresponding to a specific community type. From the perspective of aging, the aging and non-aging groups respectively corresponded to ‘aging’ and ‘non’. For population, high density and general density were respectively represented by the letters H and G. Communities dominated by high-income, middle-income, and low-income residents were represented by 1, 2, and 3, respectively. Next, the local population, mixed population, and foreign population were respectively represented by 1, 2, and 3, respectively. For example, the designation ‘Aging-H-3-2’ represents the aging, high density, low income, and mixed population community. Table 3 shows a list of community designations in Shanghai.

**Table 3:** Community types and designations in Shanghai.

Age structure	Population density	Income level	Registered residence structure	Designation
Aging	High density	High-income	Local population	Aging-H-11
			Mixed population	Aging-H-12
			Foreign population	Aging-H-13
		Middle-income	Local population	Aging-H-21
			Mixed population	Aging-H-22
			Foreign population	Aging-H-23
		Low-income	Local population	Aging-H-31
			Mixed population	Aging-H-32
			Foreign population	Aging-H-33
	General density	High-income	Local population	Aging-G-11
			Mixed population	Aging-G-12
			Foreign population	Aging-G-13
		Middle-income	Local population	Aging-G-21
			Mixed population	Aging-G-22
			Foreign population	Aging-G-23
		Low-income	Local population	Aging-G-31
			Mixed population	Aging-G-32
			Foreign population	Aging-G-33

### 3.3. Spatial pattern of community type differentiation

**Fig 2:**
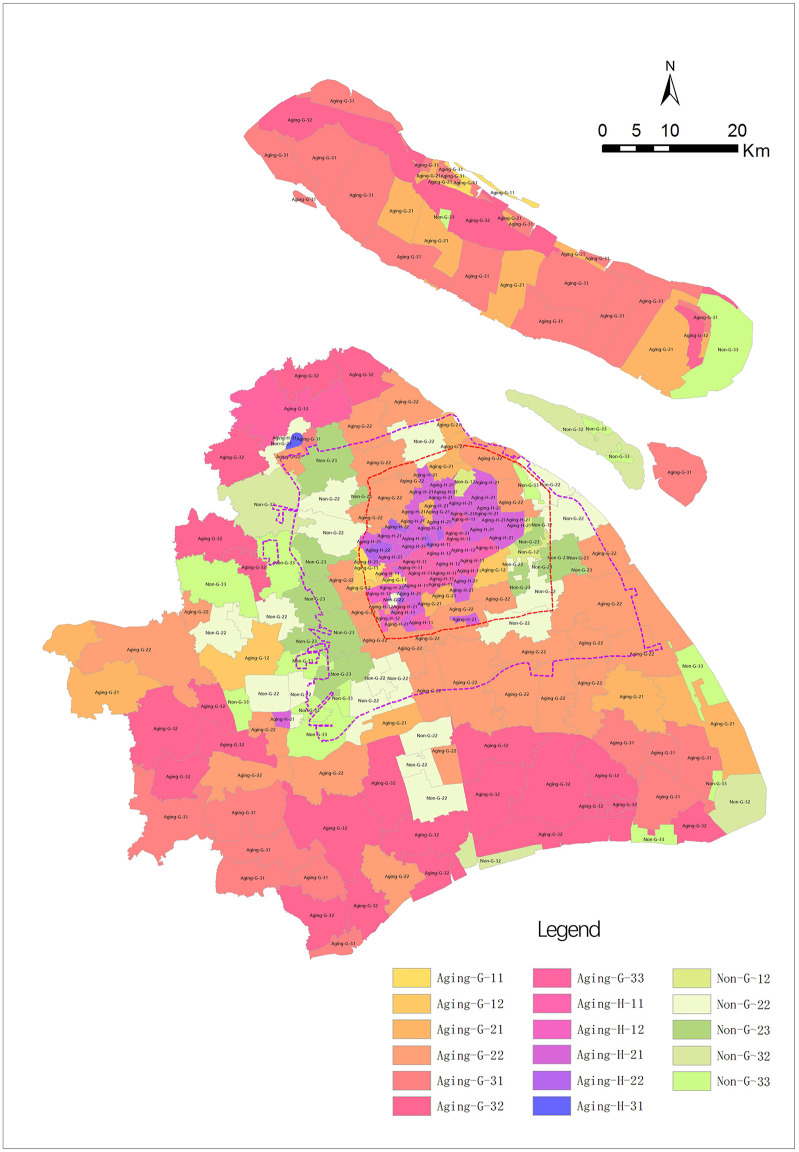
The spatial pattern of community differentiation in Shanghai. Source: Created by the author based on the base map of Shanghai from the Open Street Map(OSM) geographic data platform(https://www.openstreetmap.org/).

The four socioeconomic attributes were spatially superimposed using GIS. There were 17 community types in Shanghai, which were coded according to the above rules. A total of 17 coding samples were placed in different spatial areas (e.g., the central city, marginal area, and peripheral area) to obtain the spatial pattern of community type differentiation for urban and rural spaces in Shanghai (Fig 2).

As shown in Fig 2, the 17 community types with different socioeconomic attributes presented a clear circular structure in the urban and rural spaces of Shanghai.

There are a large number of high-density and aging communities in the central city. Among them, the central circle is mainly comprised of the Aging-H-12 type (aging, high density, high income, mixed population), with the total number of 9; most subdistricts containing the traditional public centre were located in the inner ring, including Bund Subdistrict, Nanjing East Road Subdistrict, and Lujiazui Subdistrict. The second circle is mostly comprised of the Aging-H-11 type (aging, high-density, high-income, local population), including Siping Road Subdistrict and Hunan Road Subdistrict. There were 29 of these communities, which was a relatively large number. The third circle was mostly comprised of the Aging-H-21 type (aging, high-density, middle income, local population), including Yanji New Village Subdistrict, Cao Yang Xin Village Subdistrict, and Wujiaochang Subdistrict. Here, there were 42 total communities, which was the largest number among all types. These three types were mostly distributed in the central urban area, with a small number of other types located in the middle. In addition to these three types of high-density and aging community types, the Aging-H-22 type is located in the central city, the number of which is 4, namely Baoshan Road Subdistrict, Tianmu West Road Subdistrict, Changzheng Town, and Hongmei Road Subdistrict. The central city has always been a densely populated place. At the same time, as the age structure of the original residents in the urban communities is gradually ageing, and the high-quality medical facilities in central city attract a certain amount of aging population from outside, the aging degree of the urban community is deepening. The analysis shows there are 86 high-density and aging communities in Shanghai. The above 84 subdistricts of this type are located in the central city, and the other two subdistricts of this type (Yueyang Subdistrict and Jiading Town Subdistrict) are located in the peripheral area, and none of them are located in the marginal area.

By spatial extrapolation, a large number of Aging-G-22 types (aging, general density, middle income, mixed population) were distributed at the edge of the central urban area and both inside and outside the edge area. There were 33 towns, including Sanlin Town, Xinzhuang Town, Gaoxing Town, and Dachang Town. Outside the Aging-G-22 type circle, a large number of Non-aging community types began to appear near the eastern central urban boundary and the western urban fringe boundary. There were 10 Non-G-23 types (non, aging, general density, middle income, foreign population), which were mixed with Non-G-22 types (non-aging, general density, middle income, mixed population). This shows that a large number of foreign residents have changed in this area. In addition, the registered residence structure has changed. Meanwhile, most migrant workers were young, which reduced the overall age compositions of their respective subdistricts.

This space continues to be extrapolated. There were a large number of aging-G-32 types (aging, general density, low income, mixed population) in the peripheral area. The 24 total were mostly towns, including Xinhai Town, Dongping Town, and Situan Town. The towns in the southern part of Shanghai and Chongming counties were mostly Aging-G-31 (aging, general density, low income, local population), with 22 total communities. In these peripheral areas, a large number of middle-aged and young people go out to work, while the elderly stay in their hometowns. It also triggered an increase in the proportion of the elderly population in some communities.

## 4. Spatial differentiation characteristics of accessibility to public service facilities in Shanghai

### 4.1. Measuring accessibility

Accessibility is an important concept used to evaluate spatial layouts in a variety of disciplines, including both human geography and urban and rural planning. Although there are various scholarly understandings of this concept, the focus is typically on ‘how easy it is to make people get close to facilities and services with the help of transportation and roads’ [23]. In view of significant changes in the allocation concept and mode of community public service facilities, the concept of a life circle emphasizes the convenience of public service facilities when walking. As such, the traffic mode is limited to the walking mode. Therefore, this study evaluated accessibility based on how difficult it was for residents to reach facilities by walking. While there are various ways to measure the level of accessibility, the minimum distance method is the most consistent with the concept [19]. The employed algorithm is also relatively simple and more suitable at the macro level. As such, we selected a more accurate network analysis method based on traffic network generation, with the shortest network path distance (d) used as the analysis support.

This study used a GIS system to organize the community attribute data, centroid locations of various facilities, and urban pedestrian road network onto a basic map in order to determine the population centre of gravity for each neighbourhood community unit [24]. This allowed us to break through the administrative boundary, calculate and screen out the shortest network path distance for facilities that could be accessed from each neighbourhood community unit, and finally calculate the shortest walking distance to a certain type of facility via population weighting:

$$D_{i}=\sum D_{ij}*W_{j}$$ (1)

where *D*_*i*_ is the shortest walking distance for the substrate to obtain class i facilities, D_ij_ is the shortest network path distance for neighbourhood community unit j to obtain class i facilities within the subdistrict, and W_j_ represents the proportion of the resident population of neighbourhood community unit j within the total district population. The greater the value of *D*_*i*_, the lower the accessibility level of the subclass I facilities; otherwise, the higher the value.

### 4.2. Spatial pattern of accessibility to various facilities

**Fig 3:**
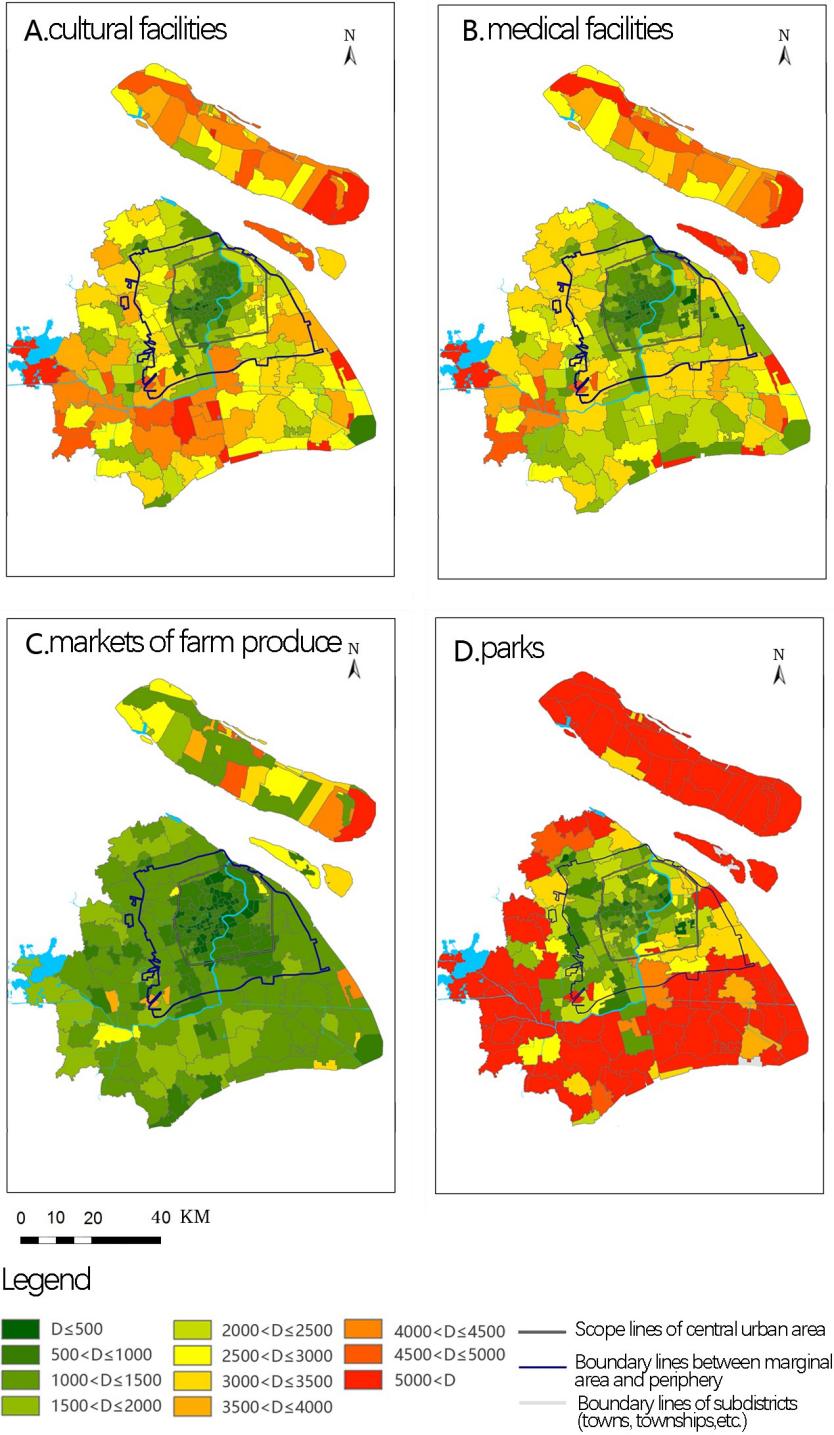
Distribution of accessibility levels of various facilities. Source: Created by the author based on the base map of Shanghai from the Open Street Map(OSM) geographic data platform(https://www.openstreetmap.org/).

Fig 3 shows the shortest walking distance to access public service facilities from each subdistrict (town, township, etc.) in Shanghai. There were clear differences in accessibility between the four types of public service facilities. However, the spatial pattern of such accessibility generally showed a tendency to decrease from the core to the periphery.

A further comparative analysis clearly showed that accessibility to cultural facilities decreased from the core to the periphery. The highest level of accessibility to cultural facilities was in the Puxi area in the central city, focusing on a large proportion of subdistricts with the shortest walking distance measuring less than 1000 m. The second highest level was found in Pudong area, with a general level of accessibility to cultural facilities in the subdistricts of marginal areas. The shortest walking distances were generally concentrated within an area 500–3000 m, which is mixed. Finally, the lowest level of accessibility to cultural facilities was found in the peripheral subdistricts, with the shortest walking distances usually being more than 3000 m, but occasionally less than 1000 m, mainly including Jiading Town Subdistrict and Shengang Subdistrict.

The spatial pattern of accessibility for medical facilities was consistent with that found for cultural facilities. In other words, accessibility decreased moving from the Puxi block, Pudong block, marginal area, and peripheral area in the central city.

The level of accessibility to farmer’s markets was significantly higher compared to other facilities. Further, there was no clear circular pattern. There was little difference between Pudong and Puxi plots in the central urban area, where the shortest walking distances were generally less than 1000 m; 48 subdistricts in the central city had walking distances of less than 500 m. The level of accessibility to farmer’s markets in subdistricts of the marginal area was slightly lower (shortest walking distances generally between 1000–1500 m). Finally, there were mixed levels of accessibility for subdistricts in the peripheral area; of these, three types had extremely low accessibility (distances greater than 2500 m) to farmer’s markets, including (1) towns in Chongming District (e.g., Gangyan Town and Chenjia Town), (2) farms (e.g., Chaoyang farm and Luchaogang farm virtual living area), and (3) industrial parks (e.g., Songjiang Industrial Park).

There were significant spatial differences in the level of accessibility to park areas, with a decreasing trend moving from the core to the periphery. There were also major differences between areas east and west of the Huangpu River. Subdistricts in the central urban and marginal areas had relatively high levels of access to park facilities, with those located to the west of Huangpu River being generally higher than those in the east. Peripheral subdistricts had very low levels of access to park areas.

### 4.3. Analysis of accessibility to various facilities based on different community attributes

#### 4.3.1 Correlation analysis between community attributes and the shortest walking distance

Taking all subdistricts in Shanghai as samples, we used IBM SPSS22.0 to examine the correlation between the shortest walking distances to the four types of facilities and the socioeconomic community attributes of each subdistrict. Table 4 shows the Pearson correlation coefficient r between the two variables.

**Table 4:** Pearson correlation coefficient between the shortest walking distance to facilities and community attributes.

	Population density	Aging level	Registered residence ratio	Income level
D(Cultural facilities)	-.675**	-.271**	-0.509**	-.642**
D(Medical facilities)	-.619**	-.256**	-.471**	-.595**
D(Farmer’s markets)	-.636**	-.100	-.377**	-.606**
D(Parks)	-.497**	.124	-0.139*	-.551**

Note:

**significant correlation at 0.01 level (bilateral);

*Significant correlation at the level of 0.05 (bilateral).

The correlation analysis revealed the four following points:

1. Population density was significantly and negatively correlated with the shortest walking distances to the four types of facilities at the 0.01 level; here, there were strong correlations with cultural facilities, medical facilities, and farmer’s markets, and moderate correlation with parks. This shows that the shortest walking distances to corresponding facilities decrease as residential population density increases. In other words, subdistricts with high population densities have good accessibility.
2. There was no correlation between the level of ageing and the shortest walking distance to farmer’s markets and parks, but the level of aging was significantly correlated with the shortest walking distance to cultural facilities and medical facilities, at the 0.01 level. Here, there were weak negative correlations between the level of aging and both cultural facilities and medical facilities. This indicates that the shortest walking distance from the subdistricts to these two types of facilities decreases with increased aging, thus highlighting a justice tendency involving these types of facilities and the elderly population.
3. The minimum distance between the registered residence and the three types of facilities (including farmer’s markets, medical facilities, and cultural facilities) was significantly related at 0.01 level. Cultural facilities and medical facilities were negatively correlated, while the farmer’s markets were correlated weakly and negatively. This shows that access to facilities increases with higher population concentrations, and that the proportion of registered residences was significantly related to the shortest walking distance to park areas at the 0.05 level (very weak correlation). This also reflects a direct relationship between the park configuration, construction results, and registration data.
4. The income level was significantly and negatively correlated with the shortest walking distance to the four types of facilities at the 0.01 level. Here, the shortest walking distance to facilities decreased corresponding to areas with higher house prices. In other words, higher housing prices were associated with better facility accessibility.

Generally speaking, there was a significant and negative correlation at the 0.01 level for population, income level, and the shortest walking distance to facilities. Further, aging level and registered residence ratio were partly related to the shortest walking distances to the facilities.

#### 4.3.2. Analysis of the mean values of accessibility to facilities in different types of communities

We counted the mean values of the shortest walking distance of the four types of public service facilities in 17 community types in Shanghai, as shown in Table 5. The mean values of accessibility to the four types of facilities public service facilities differed among different community types.

**Table 5:** The mean values of the accessibility of different types of communities.

Serial number	Community type	D(Cultural facilities)	D(Medical facilities)	D(Farmer’s markets)	D(Parks)
1	Aging-H-31	733	460	523	371
2	Aging-H-12	837	838	521	1225
3	Aging-H-11	696	844	547	1281
4	Aging-H-21	939	876	558	1207
5	Aging-H-22	914	1137	670	1468
6	Non-G-23	2843	1079	1543	1234
7	Non-G-12	2039	1906	1721	2508
8	Non-G-22	2448	1987	1177	2215
9	Aging-G-33	3001	2476	2162	4783
10	Aging-G-12	2570	2706	1302	3302
11	Aging-G-22	2539	2777	1375	4374
12	Non-G-32	4311	3242	2979	10202
13	Aging-G-21	2625	4190	1528	8613
14	Aging-G-32	3515	4229	2109	13761
15	Aging-G-11	2100	4242	1435	9563
16	Aging-G-31	3144	6038	2452	29261
17	Non-G-33	4661	13222	2414	28579

The mean values of accessibility to the four types of facilities generally showed higher levels in the five types of Aging-H-31, Aging-H-12, Aging-H-11, Aging-H-21, and Aging-H-22, which are high-density and aging communities. Among them, the Aging-H-31 type (aging, high-density, low-income, local population) community has the highest level of facility accessibility, typically represented by Jiading Town Subdistrict located in the peripheral area, which relies on Jiading Old Town to form a well-equipped and highly densely populated community. Although the other four categories differ in social attributes such as household registration structure and income level, they are all located within the central city and uniformly show the characteristics of high-density population gathering and an aging structure of residents.

The mean values of accessibility to the three types of facilities (cultural facilities, medical facilities, and parks) show moderate levels in 6 types of communities, namely Non-G-23, Non-G-12, Non-G-22, Aging-G-33, Aging-G-12, and Aging-G-22, while the mean values of accessibility of communities show the lowest levels in the other 6 types. The mean values of accessibility to these three types of facilities show a relatively consistent distribution among different types of communities. However, the mean accessibility values of Farmer’s markets did not show a significant difference pattern among these 12 types of communities.

### 4.4. Spatial matching characteristics of community population distribution and facility accessibility

**Fig 4:**
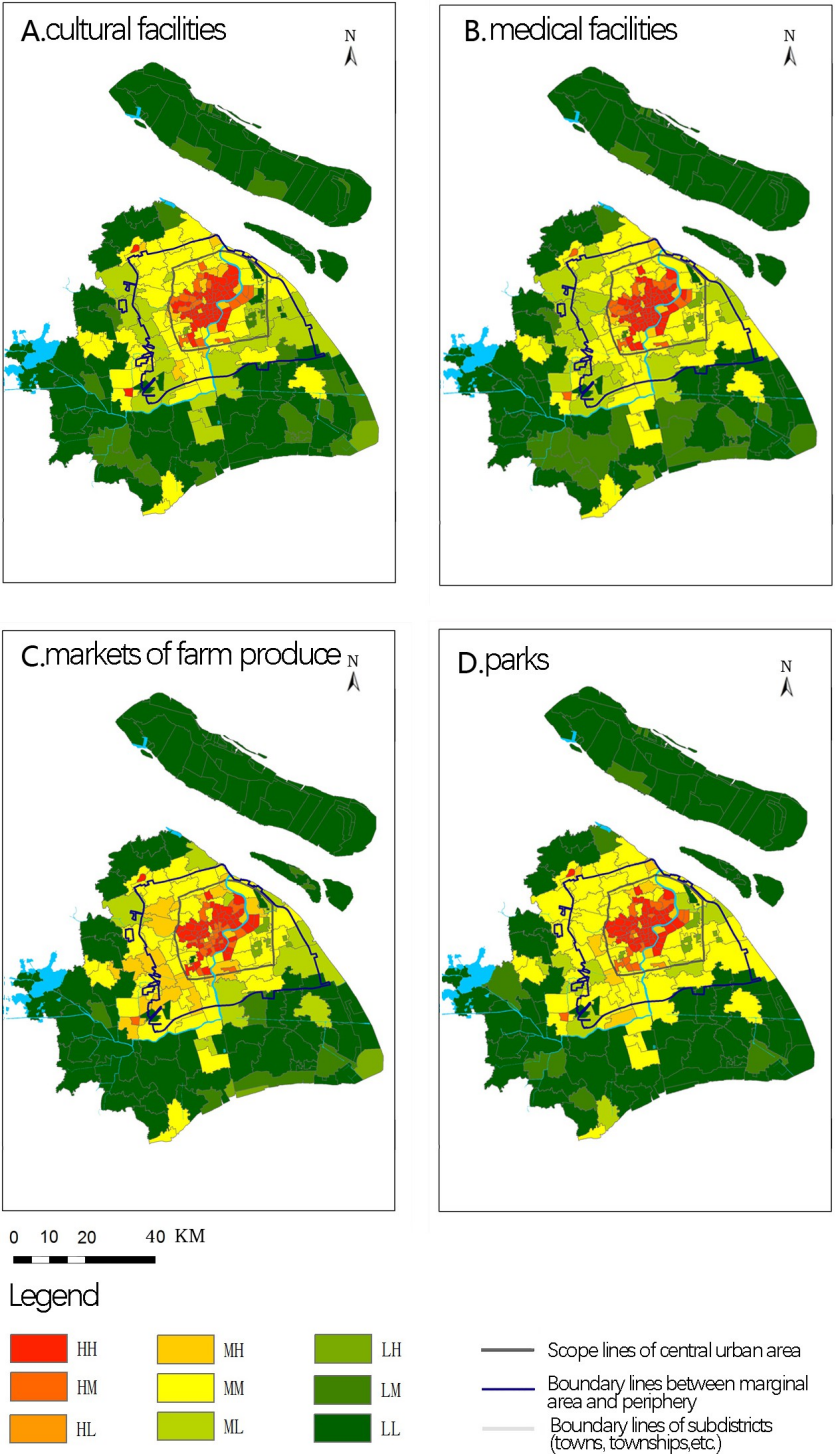
The spatial pattern of matching values between population density and the accessibility of various facilities. Source: Created by the author based on the base map of Shanghai from the Open Street Map(OSM) geographic data platform(https://www.openstreetmap.org/).

Based on the above analysis, we selected population density and the shortest walking distance to facilities for trisection spatial matching. Using the equal quantity classification method, the residential population density of each subdistrict (town, township, etc.) in Shanghai was divided into three categories, including high, medium, and low. The shortest walking distance to facilities available to each subdistrict (town, township, etc.) was divided into three categories, including high, medium, and low accessibility. According to the classifications for both population density and accessibility, this was further divided into nine types, including the double high (HH), high density and medium accessibility (HM), high density and low accessibility (HL), medium density and high accessibility (MH), double medium (MM), medium density and low accessibility (ML), low density and high accessibility (LH), low density and medium accessibility (LM), and double low (LL). Next, GIS was used to spatially match community population density and facility accessibility (Fig 4).

Generally speaking, the spatial matching of population density and facility accessibility presented both a circular pattern and heterogeneity. The matching of HH, MM, and LL showed obvious circular distribution characteristics moving from the central urban area to the outside. The HH subdistricts were concentrated in the Puxi area of the central urban area and the riverside area of Pudong area. The MM subdistricts are mainly continuously distributed in the marginal areas, while the LL subdistricts were mainly distributed in peripheral cities and towns. At the same time, there was a partial heterogeneous distribution in the peripheral areas. The Jiading Town and Yueyang districts had HH matching for multiple types of facilities. The Petrochemical subdistrict, Huinan Town, and Yingpu showed MM matching for multiple types of facilities, while the Shengang, Jinqiao Export Processing, and Zhangjiang High Tech Parks also had LH characteristics. Various facilities also presented different matching realities. Among these, the circular structure of spatial matching between population density and farmer’s market accessibility was slightly weaker than that of other facilities. There were no HL or LH subdistricts.

Combined with the spatial distribution of community type differentiation, it can be seen that there is a double medium-sized distribution matching for population density and facility accessibility (Table 6) in the planned suburban and central suburban new towns. The subdistricts where new towns were located showed the same socioeconomic attributes, all of which were of the Non-G-22 type (non-aging, general density, middle income, mixed population).

**Table 6:** The matching value of population density and community accessibility to facilities in planned new towns.

Type	Subdistrict name	New town	Population- medical facility	Population-farmer’s markets	Population-park	Population- cultural facility
Non-G-22	Jvyuan Subdistrict	Jiading new town	MM	MM	MM	MM
	Yangxing Town	Baoshan new town	MM	MM	MM	MM
	Xiayang Subdistrict	Qingpu new town	MM	MM	MM	MM
	Fangsong Subdistrict	Songjiang new town	ML	MM	MM	MM
	Zhongshan Subdistrict		MM	MM	MM	MM
	Zhuanqiao Town	Minhang new town	MM	MM	MM	MM
	Nanqiao Town	Nanqiao new town	MM	MM	MM	ML
Aging-G-32	Wei Town	Jinshan new town	LL	LM	LL	LL
Non-G-32	Shengang Subdistrict	Lingang new town	LM	LM	LL	LH
Aging-G-31	Chengqiao Town	Chengqiao new town	LM	LM	LM	LM

Note: The first word of the matching value represents the gradient of population density, while the second letter represents the gradient of accessibility. L, M, and H respectively represent low, medium, and high.

## 5. Mechanism analysis

We selected the common and extreme phenomena of community type and facility accessibility spatial distribution to analyze the mechanism behind the formation of facility accessibility spatial patterns from the perspective of community type differentiation.

### 5.1. Continuity of urban-rural patterns and differences in allocation norms

There were obvious differences in the socioeconomic compositions and spatial scales between urban and rural areas. The polarized pattern of population distribution between these areas has persisted for a long time. The central city of Shanghai has remained an area of high population density that continues to attract a large number of foreign migrants. In Shanghai, urban spatial spread and expansion are influenced by many factors, including population policy, planning guidance, and the market economy. Migrant populations have moved to the urban fringes, where a large number of workers with limited economic capacities are now concentrated. At the same time, large-scale affordable housing has been planned and arranged in the suburbs to accommodate substantial urban renewal and resettlement, thereby resulting in many medium-density communities. Meanwhile, peripheral areas with low levels of urbanization are mostly occupied by towns and townships with no prominent central roles, a limited number of migrants, and high distribution of low-density communities. In general, there are obvious differences in population distribution between urban and rural areas, and there is a long-term gradient difference in population density from the central city to the peripheral areas. The urban-rural pattern has historical continuity and will continue to persist for a long time. At the same time, the construction standards and norms guiding the allocation of community public service facilities in Shanghai have differed between communities in different locations for a long time. Both the differential allocation according to urban and rural areas and different locations have resulted in uneven levels of facility accessibility between urban and rural spaces.

As shown in Fig 4, the spatial matching between population density and facility accessibility revealed differential levels of access. However, the three types of high-level matching still reflect the beneficial principle of urban-rural pattern continuation and public service facility allocation. Both the gradient difference in urban-rural population density and the classification guidance of public service facility allocation standards have produced a circular distribution consisting of HH, MM, and LL matching in urban and rural spaces.

### 5.2. Continuity of population agglomeration and accumulation of facility construction

After comparing the urban-rural spatial pattern of community type differentiation (Fig 2) and spatial distribution of HH subdistricts (Fig 4), this study found that HH matching subdistricts in the central city mainly include the two following types: (1) Many Aging-H-12 (aging, high density, high income, mixed population) communities in the core area of the central city, including Bund Subdistrict, Nanjing East Road Subdistrict, and Lujiazui Subdistrict. This is mainly due to gentrification of the urban central area in the context of inner-city renewal in Shanghai. Under joint action of the government and market forces, a large number of original residents are filtered out, leaving a high-income area; (2) Aging-H-11 (aging, high density, high income, local population) and Aging-H-21 (aging, high density, middle income, local population) were the two types. Although there were certain differences in income levels, all contained household registration populations.

As a highly mature urban area, Shanghai’s urban and community construction has always been at the national forefront. The accumulation of urban public service facilities has a high overall level of facility supply. However, there are also differences in the number and grade of facilities between different urban areas, with some differences in facility accessibility. Since the industrial revolution of the 19th century, Shanghai has built a central urban area around the Bund in the Puxi area, which contains a large number of high-quality commercial, cultural, medical, and other facilities; the hierarchical system is perfect. As a result, there is generally good facility access in the Puxi area, especially when compared to the Pudong area. In the process of urban space expansion, the planned new town in the fringe area is also equipped with high-level public service facilities in order to attract population agglomeration. In these areas, facility accessibility was also significantly higher than in other suburban areas. There were few high-level public service facilities in the peripheral areas, with a less efficient configuration in the central city.

It can be seen that A large number of HH communities have formed in the central city under the joint action of high population agglomeration and facility accumulation, mainly including gentrified Aging-H-12 communities in the core area as well as Aging-H-11, Aging-H-21, and other communities. The subdistrict of Jiading town was the only community in the peripheral area with Aging-H-31 (aging, high density, low income, local population). There was an HH matching type for population density and community accessibility. The formation mechanism is the joint action of the populated old city (old urban area of Jiading) and internal facilities.

### 5.3. Urban planning guidance and the population agglomeration effect

According to the 1966 Shanghai urban system planning goal, the 12th Five Year Plan specifies that Shanghai will focus on developing three types of nine new towns. This includes suburban new towns (Minhang new town and Baoshan new town), central suburban new towns (Qingpu new town, Jiading New Town, Songjiang New Town, and Nanqiao new town), and suburban new towns (Lingang New Town, Jinshan new town, and Chongming Chengqiao new town). To guide the population to gather in the planned new town, the government provides policy support in many aspects, such as capital and population. The suburban New Town and central suburban new town have developed quite rapidly, with good population agglomeration and facility configuration; the matching value between population density and facility accessibility was MM (Table 6). Further, the subdistricts under the jurisdiction of the new town showed consistent socioeconomic attributes. They were of the Non-G-22 type (non-aging, general density, middle income, mixed population). Compared with the construction results of nine new towns, the matching values of facility accessibility and community population density were significantly different. On the other hand, there was insufficient population agglomeration in the three new towns in the outer suburbs, with population density concentrated in the low-value area. Under the joint actions of urban planning guidance for new town construction, population agglomeration, and location selection, both the suburban new towns and central suburban new towns showed a double and medium-sized distribution of the same community type.

## 6. Discussion

This paper adopts a scientific and clear classification, which is helpful in understanding the developmental state and process of the community, while the classification of community types is helpful in better understanding the community. The selection of the subdistrict level for community type division is consistent with the current administrative unit division in regional scope, which has guiding significance for the spatial layout and implementation of public service facilities. This paper focuses on the spatial distribution of four public service facilities and reveals the spatial model and correlation from the ‘core’ of Shanghai to the surrounding suburbs and quasi-rural areas. The paper provides strong evidence based on rigorous methods to support its discovery.

Compared with the previous research, the research scope of this article has a breakthrough, breaking through the limitation of the urban area, thus expanding to the urban and rural areas. Due to the choice of the analysis scope is too large, the analysis unit of this article also has certain limitations. The community statistical unit corresponding to this article is the administrative division rather than the construction land range, so it triggers the differences in the socio-economic attributes within the subdistricts. While the community type of Shanghai does not represent the general situation of all big cities in China, it does reflect the common differences of community types in China’s mega cities. China is undergoing rapid urbanization, and the position and influence of Chinese cities in the world urban system are gradually increasing. As a typical representative of mega cities, the spatial evaluation of community differentiation and accessibility to public service facilities in Shanghai deepens the practical significance of the research. At the same time, this study enriches the research cases of Asian cities in the spatial dimension, and has reference significance for international cities.

Public service facility allocation is a complex problem that must be considered from the dimensions of the distribution system, spatial layout, social fairness, and justice. Based on objective data, this study evaluated the spatial distribution of accessibility to public service facilities in Shanghai and conducted facility allocation based on the structural characteristics of the target population according to community type differentiation. The ‘people-oriented’ spatial allocation model of public service facilities must face the reality that community type differentiation exists, and will continue to do so for a long time. As such, we should comprehensively evaluate more micro-level and detailed problems. Future studies should analyze facility quality, arrival cost, resident behavioural characteristics, and demand differences.

## 7. Conclusion

This study produced three main findings. First, there were significant differences between community types in Shanghai. In this regard, four types of socioeconomic attributes (population density, age structure, registered residence structure, and income level) were superimposed, resulting in 17 different types. These communities exhibited a circular structure in urban and rural spaces in Shanghai.

Second, there were some differences in the level of accessibility to the four types of public service facilities at the municipal level, with a generally decreasing characteristic moving from the core to the periphery. A correlation analysis between community attributes and the shortest walking distance to the four types of public service facilities showed that population density, income level, and the shortest walking distance to facilities were significantly and negatively correlated at the 0.01 level. Aging level and registered residence ratio were partly or unrelated to the shortest walking distance to facilities. Meanwhile, the mean values of the shortest walking distances of the four types of public service facilities showed that the mean values of accessibility vary among different community types.

Third, the spatial matching of population density and facility accessibility presented circles and heterogeneity. The three types of extreme matching showed an obvious circular distribution. The HH type was mostly concentrated in the Puxi area of the central urban area and the riverside area of the Pudong area, while the MM type was mostly distributed in the urban fringe, and the LL type was mainly distributed in the urban periphery. At the same time, the peripheral area showed a partial heterogeneous distribution, and the planned suburban and central suburban new towns showed double-and medium-sized matchings of the same community type (Non-G-22). The mechanism of formation behind the spatial distribution of accessibility to public service facilities from the perspective of community type differentiation is the continuity of urban-rural patterns and the differences in allocation norms, the continuity of population agglomeration and the accumulation of facility construction, the guidance of urban planning, and the effect of population agglomeration.
